# Individual and Organisational Determinants Associated with Maintenance Tocolysis in the Management of Preterm Labour: A Multilevel Analysis

**DOI:** 10.1371/journal.pone.0050788

**Published:** 2012-12-13

**Authors:** Caroline Diguisto, Camille Le Ray, Françoise Maillard, Babak Khoshnood, Eric Verspyck, Franck Perrotin, François Goffinet, EVAPRIMA group

**Affiliations:** 1 INSERM UMR S953, Epidemiological Research Unit on Perinatal Health and Women’s and Children’s Health, Pierre et Marie Curie University, Paris, France; 2 Department of Obstetrics and Gynaecology, Port-Royal Maternity, Cochin Saint-Vincent-de-Paul Hospital, Assistance Publique des Hopitaux de Paris, Université Paris Descartes, Sorbonne Paris Cité, Paris, France; 3 Department of Obstetrics and Gynaecology, University Hospital of Rouen, Rouen, France; 4 Department of Obstetrics and Gynaecology, University Hospital of Tours, François Rabelais University, Tours, France; 5 Premup Foundation, Paris, France; Hospital Clinic, University of Barcelona, Spain

## Abstract

**Background:**

Clinical guidelines do not recommend maintenance tocolysis for the management of preterm labour. The French national survey EVAPRIMA revealed it was administered to more than 50% of women hospitalised for preterm labour. Our aim was to identify the individual and organisational determinants associated with maintenance tocolysis.

**Methods:**

The study was a secondary analysis of the prospective population-based EVAPRIMA study database. Population study included every women hospitalised for preterm labour and at risk of receiving maintenance tocolysis, over a one month period, in 99 randomly selected French maternity units. **Main outcome** was the prescription of maintenance tocolysis. The association between maintenance tocolysis and individual (maternal or obstetrical) and organisational determinants were evaluated with multilevel analysis.

**Results:**

Of the 531 women included, 68.9% (95% CI 0.65–0.73) received maintenance tocolysis. The only individual factor associated with maintenance tocolysis was gestational age at admission; the rate of maintenance tocolysis was higher among women hospitalised before 32 weeks of gestation. The significantly different rates between maternity units demonstrated the existence of a maternity unit effect. Maintenance tocolysis was also associated with organisational determinants and was more frequent in level 1 (ORa = 6.54[2.21–19.40]) and level 2 maternity units (ORa = 3.68[1.28–10.59]), in units with less than 1500 deliveries/year (ORa = 5.27[4.43–19.44]), and in specific areas of France.

**Conclusion:**

A maternity unit effect, explained partly by the organisational characteristics of the units, plays a major role in the practice of maintenance tocolysis. Widespread dissemination of these results might improve adherence to clinical guidelines.

## Introduction

Preterm delivery is a leading cause of perinatal mortality and morbidity in industrialised countries [1]. Worldwide prevalence of preterm delivery ranges from 6 to 15%; the rate in France is 7.2%, and two thirds of these preterm deliveries are spontaneous and preceded by preterm labour [2], [3].

By postponing delivery, acute tocolysis allows the transfer of the patient to a unit with a suitable neonatal ward and the administration of corticosteroids, thereby decreasing neonatal morbidity and mortality [4], [5]. Acute tocolysis usually lasts 48 hours. Maintenance tocolysis is broadly defined as the continuation of tocolytic treatment after 48 hours and after acute tocolysis has been clinically effective.

Because of the absence of evidence of any benefit from maintenance tocolysis and the possibility of maternal or fetal side effects, in 2002, neither the National College of French Gynaecologists and Obstetricians (CNGOF) nor the Royal College of Obstetricians and Gynaecologists (RCOG) recommends the practice of maintenance tocolysis in their clinical practice guidelines [6], [7]. Nonetheless, the EVAPRIMA (Enquête Française sur la Prise en charge des Menaces d’Accouchement Prématuré) study, examining the management of preterm labour in a representative sample of French maternity units three years after the publication of the CNGOF guidelines, showed surprisingly that 54.3% of women hospitalised for preterm labour received maintenance tocolysis [8]. EVAPRIMA was a national observational practice survey. It offered the opportunity to study organisational determinants for each participating maternity unit and individual determinants for every women included in the study.

Contrary to practice survey with questionnaires sent to physicians which induce many biases, this observational survey and its database provided a unique opportunity to assess the frequency of this unrecommended practice and the associated determinants.

We performed a secondary analysis of the EVAPRIMA database to identify individual and organisational determinants associated with maintenance tocolysis to attempt to understand why this practice remains so common in France despite the guidelines. Our aim was also to publicize these results among physicians to improve guideline adhesion.

**Figure 1:**
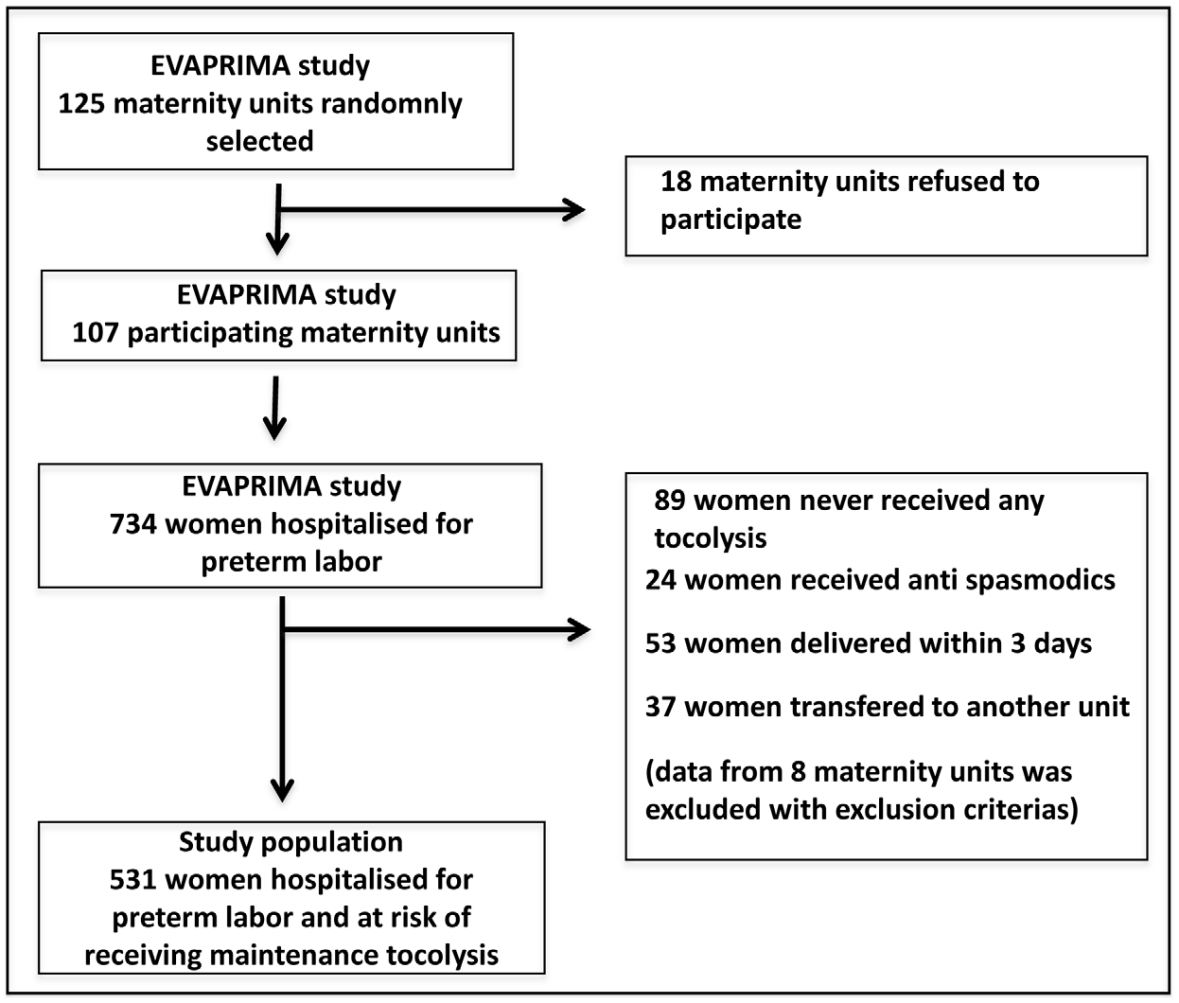
Population study.

## Methods

### Data Source

The EVAPRIMA study was a national prospective observational survey, conducted during the month of May, 2005. This population-based study examined clinical practices regarding management of women admitted for preterm labour in a representative sample of French maternity units. The National Data Protection Authority and the Institutional Review Board of Cochin Hospital (in a decision dated 15 April 2005) both approved this study.

To obtain a representative sample of maternity units, we randomly selected 20% of the 614 French maternity units, after stratification for level of perinatal care, maternity unit status (private or public) and geographical area. Investigators in the participating units included every woman hospitalised that month for preterm labour between 22 and 36 weeks of gestation, whether the pregnancy was singleton or multiple and whether membranes were intact or ruptured. Exclusion criteria were direct admission to the delivery room for delivery, stillbirth, and participation in a clinical trial in the previous month. Data were prospectively collected by a local investigator. The national co-ordinator visited 11 randomly selected units to check the completeness and validity of data collection. Details on the methods have been published previously [8].

### Study Population

The EVAPRIMA study population included 734 women from 107 maternity units. We selected from them the women eligible for maintenance tocolysis, that is, those who might have received it. The exclusion criteria were: women who had not received any tocolysis during hospitalisation (n = 89), women who delivered within three days of admission, for whom acute tocolysis was considered a failure (n = 53), and women whose subsequent tocolytic treatment was unavailable because they were transferred to another maternity unit (n = 37). Thus, our study population included 531 women from 99 maternity units [Figure 1].

### Outcome Factors and Other Data Collected

The main outcome measure was a binary variable of maintenance tocolysis (yes/no). Information about any such treatment included date it began, type of tocolytic drug, route of administration, and gestational age at which treatment stopped.

Two categories of explanatory factors were considered: individual characteristics of women and organisational characteristics related to the health care facility. At the individual level, the characteristics studied included the mother’s age, any history of late miscarriage or preterm delivery, parity, multiple pregnancies, cervical cerclage and clinical criteria at admission, including gestational age, Bishop score, frequency of uterine contractions, sonographic cervical length, and membrane status. Two composite variables were created: “high risk pregnancy” for women with a history of preterm delivery, late miscarriage, multiple pregnancy, or cervical cerclage, and “high risk at admission” for women with a Bishop score higher than 4, a sonographic cervical length shorter than 25 mm, more than six uterine contractions per 30 minutes or ruptured membranes.

The organisational characteristics studied were level of perinatal care, legal status (public or private), size of maternity unit and geographical area. Level of perinatal care was defined by official French regulations on the safety of childbirth [9], [10]: level 1 units have no neonatal ward and are not required to have a paediatrician onsite; level 2 units have neonatology facilities to manage infants born at 32 weeks of gestation or later and require the presence of a paediatrician during the day and either their presence or on-call availability at night and weekends; and level 3 units have an onsite neonatal intensive care unit and a neonatologist onsite 24 hours a day, seven days a week. Maternity unit size was defined by the number of deliveries per year: less than 1500, 1500–2500, more than 2500. For geographic area, France was divided in five areas: Ile de France, Northwest, Northeast, Southeast and Southwest.

### Statistical analysis

Tocolytic agents and duration of maintenance tocolysis were described. We used univariate and multivariate analysis to examine the associations between individual and organisational characteristics and maintenance tocolysis. For both univariate and multivariate analysis we used multilevel models, i.e., two-level hierarchical logistic regression models which took into account the hierarchical structure of data, with women (level 1 =  individual level) nested within maternity units (level 2 =  organisational level).

Multivariate analyses were conducted with 3 sets of models. First we estimated an empty (or null) model, a random intercept model with no predictor variables (model 1) to obtain the baseline hospital-level variance (var^(1)^) and to determine the existence of any “maternity unit effect” for the practice of maintenance tocolysis. Next, in a second model (model 2), we included women’s individual characteristics as predictor variables. This model allowed us to investigate the association between frequency of maintenance tocolysis and individual-level variables and to estimate the residual maternity-unit variation after adjustment for the individual–level variables (var^(2)^). In a third series of models we added organisational characteristics as predictor variables after adjustment for individual-level variables: level of perinatal care (model 3a), size of the maternity unit (model 3b), and geographic area (model 3 c) (var^(3)^). Organisational characteristics that were not independent according to a chi-square test were not simultaneously entered in any model, to avoid correlation. Therefore, only level of perinatal care and geographical region were simultaneously included as predictor variables in a model (model 3d). We used the proportional change in the variance (PCV) with PCV =  [var^(2)^-var^(3)^/var^(2)^], to assess the extent to which differences between units in their practices of maintenance tocolysis might be explained by organisational characteristics in each model. As stated by the formula, the reference variance used to calculate the PCV was the variance of model 2, which was adjusted for women’s individual characteristics.

Variables for which more than 15% of the data was missing were not included in our multivariate analysis, i.e. only one variable (sonographic cervical length at admission). We added a missing data class to the variables for which the percentage of missing data was between 5 and 15%. The statistical analysis used Stata 9.1 software (StataCorp, College Station, TX, USA).

## Results

Of the 531 women included, 68.9% (95% CI 0.65–0.73) received maintenance tocolysis. Its median duration was 37 days (interquartile range (ICQ): 24–46). The most frequently used agent was Nicardipine (43%), followed by Beta-agonists (33%) and Nifedipine (23.5%). Oxytocin receptor antagonists were never used for maintenance tocolysis in our study.

Table 1 summarises the characteristics of the study population, among whom 23% had a “high risk pregnancy” and 74% were considered at “high risk at admission”. Finally, 30.7% (n = 160/531) of the women hospitalised for preterm labour delivered preterm.

**Table 1:** Characteristics of the study population.

	N = 531n (%)
Maternal age (years)	
<25	179 (33.7)
25–35	299 (56.3)
>35	53 (10)
Nulliparas	310 (58.4)
History of late miscarriage	24 (4.5)
History of preterm delivery	48 (9)
Multiple pregnancy	60 (11.3)
Cervical cerclage	15 (2.8)
High risk pregnancy *	121 (22.8)
>34	43 (8.1)
32–34	149 (28.1)
≤32	339 (63.8)
Bishop score at admission	
≤4	320 (60.2)
5–6	106 (20)
>6	44 (8.3)
Missing data	61 (11.5)
Number of uterine contractions at admission	
≤6 per 30 minutes	292 (55.6)
>6 per 30 minutes	219 (41.2)
Sonographic cervical length at admission (mm)	
≤25	167 (31.4)
>25 & ≤30	54 (10.2)
>30	60 (11.3)
Missing data	250 (47.1)
Ruptured membranes at admission	26 (4.9)
High risk at admission **	392 (73.8)
Level of perinatal care	
−1	170 (32.0)
−2	215 (40.5)
−3	146 (27.5)
Legal status	
Public	360 (67.8)
Private	171 (32.2)
Size of maternity ward (deliveries/year)	
<1500	299 (56.3)
1500–2500	123 (23.1)
>2500	109 (20.6)
Geographic area	
Ile de France	154 (29.0)
Northwest	72 (13.5)
Northeast	136 (25.7)
Southeast	108 (20.3)
Southwest	61 (11.5)

* High risk pregnancy : History of late miscarriage, preterm delivery, multiple pregnancies or cervical cerclage.

** High risk at admission: Bishop score >4, >6 uterine contractions per 30 minutes, ruptured membranes at admission.

As shown in Table 2, in the univariate analyses, the rate of maintenance tocolysis was higher among women with intact membranes than among those with ruptured membranes (69.9% vs 50.0%; p = 0.03). The rate of maintenance tocolysis was also significantly higher for women admitted before 32 weeks of gestation compared with those admitted between 32 and 34 weeks or after 34 weeks (70.5% vs 69.8% vs 53.5%; p = 0.07). Other individual factors, including the composite variables of “high risk pregnancy” and “high risk at admission”, were not significantly associated with maintenance tocolysis.

**Table 2:** Association between individual determinants and maintenance tocolysis (univariate analysis).

	Maintenance tocolysisYes (%)	Crude OR95% CI	p
Maternal age (years)			
<25 (n = 179)	118 (65.9)	1.00	0.11
25–35 (n = 299)	205 (68.6)	0.95 (0.57–1.58)	
>35 (n = 53)	43 (81.1)	1.70 (0.67–4.27)	
Parity			
nulliparas (n = 310)	211 (68.1)	1.00	0.61
multiparas (n = 221)	155 (70.1)	1.06 (0.66–1.72)	
History of late miscarriage			
No (n = 507)	349 (68.8)	1.00	0.84
Yes (n = 24)	17 (70.8)	1.41 (0.47–4.30)	
History of preterm birth			
No (n = 483)	332 (68.7)	1.00	0.77
Yes (n = 48)	34 (70.8)	1.73 (0.72–4.14)	
Type of pregnancy			
singleton (n = 471)	326 (69.2)	1.00	0.69
multiple pregnancies (n = 60)	40 (66.7)	1.39 (0.32–6.02)	
Cervical cerclage			
no (n = 516)	356 (69.0)	1.00	0.85
yes (n = 15)	10 (66.7)	0.78 (0.38–1.60)	
High risk pregnancy*			
no (n = 410)	282 (68.8)	1.00	0.89
yes (n = 121)	84 (69.4)	1.18 (0.67–2.08)	
Gestational age at admission (weeks of gestation)			
>34 (n = 43)	23 (53.5)	1.00	0.07
32–34 (n = 149)	104 (69.8)	1.95 (0.79–4.84)	
≤32 (n = 339)	239 (70.5)	3.02 (1.27–7.14)	
Bishop score at admission			
≤4 (n = 320)	220 (68.8)	1.00	0.83
5–6 (n = 106)	72 (67.9)	1.02 (0.56–1.89)	
>6 (n = 44)	33 (75.0)	1.12 (0.46–2.75)	
Missing data	41 (67.2)	0.71 (0.32–1.56)	
Uterine contractions per 30 minutes at admission			
≤6 (n = 295)	212 (67.9)	1.00	0.56
>6 (n = 219)	154 (70.3)	0.94 (0.57–1.56)	
Membrane status at admission			
Intact (n = 505)	353 (69.9)	1.00	0.03
Ruptured (n = 26)	13 (50)	0.23 (0.08–0.63)	
High risk at admission**			
no (n = 188)	93 (66.9)	1.00	0.54
yes (n = 343)	273 (69.6)	0.91 (0.55–1.51)	

As Table 3 shows, maternity-unit characteristics significantly influenced the rate of maintenance tocolysis in the univariate analysis. The maintenance tocolysis rate was higher among women who had been hospitalised in level 1 and 2 maternity units than in level 3 units (76.5% vs 69.8% vs 58.9%; p<0.01). It was also higher in units with less than 1500 deliveries per year than in those with either 1500 to 2500 or more than 2500 (74.6% vs 59.4% vs 64.2%; p<0.01). Maintenance tocolysis was also more frequent in two regions – the Ile de France and the Southwest. Maternity unit’s legal status was not significantly associated with the practice of maintenance tocolysis.

**Table 3:** Association between organisational determinants and maintenance tocolysis (univariate analysis).

	Maintenance tocolysisYes (%)	Crude OR (95% CI)	p
Level of perinatal care			
1 (n = 170)	130 (76.5)	4.84 (1.60–13.63)	<10^−2^
2 (n = 215)	150 (69.8)	3.05 (1.15–10.56)	
3 (n = 146)	86 (58.9)	1.00	
Legal status			
Public (n = 360)	239 (66.4)	1.00	0.07
Private (n = 171)	127 (74.3)	1.73 (0.76–3.93)	
Size of maternity ward (deliveries/year)			
<1500 (n = 299)	223 (74.6)	3.91 (1.12–13.71)	<10^−2^
1500–2500 (n = 123)	73 (59.4)	1.56 (0.38–6.37)	
>2500 (n = 109)	70 (64.2)	1.00	
Geographic area			
Ile de France (n = 154)	128 (83.1)	6.56 (2.09–20.48)	<10^−3^
Northwest (n = 72)	42 (58.3)	1.29 (0.40–4.17)	
Northeast (n = 136)	89 (65.4)	2.75 (0.98–7.72)	
Southeast (n = 108)	57 (52.8)	1.00	
Southwest (n = 61)	50 (81.9)	4.45 (1.22–16.20)	

In the multivariate analyses, the study of the empty (or null) model showed a significant “maternity unit effect” for the practice of maintenance tocolysis, with the rate varying significantly from one maternity unit to another (p<0.001). Table 4 reports the results of the multilevel analysis, which includes the adjusted odds ratios between maintenance tocolysis and individual and organisational determinants. It also includes the calculation of the proportional change in the variance (PCV) for each model, to assess the extent to which differences in maintenance tocolysis practices might be explained by organisational characteristics. Model 2, which only had women’s individual characteristics as predictor variables, had a reference variance of 2.42. The third series of models, adjusted both for individual characteristics and for organisational characteristics, i.e., level of perinatal care, size of the maternity unit and geographical area, allowed us to calculate the PCV. When we adjusted for women’s individual characteristics, the unit’s level of care, and geographic region (model 3d), variance decreased to 1.54, and the PCV was 0.38, i.e., 38% of the residual variance could be explained by level of perinatal care and geographic area. In this model, the risk of receiving maintenance tocolysis in level 1 and level 2 maternity units was 6.54 (95% CI 2.21–19.40) and 3.68 (95% CI 1.28–10.59) times higher, respectively, than in level 3 units. The risk of receiving maintenance tocolysis was as much as 6.83 (95% CI 2.20–21.16) times higher in specific regions.

**Table 4:** Association between individual and organisational determinants and maintenance tocolysis (multivariate analysis).

	Model 2OR (95% CI)	Model 3aOR (95% CI)	Model 3bOR (95% CI)	Model 3cOR (95% CI)	Model 3d
Maternal age (years)					
<25	1.00	1.00	1.00	1.00	1.00
25–35	0.86 (0.50–1.46)	0.82 (0.48–1.40)	0.88 (0.52–1.49)	0.88 (0.52–1.49)	0.84 (0.49–1.43)
>35	1.56 (0.60–4.05)	1.55 (0.60–3.99)	1.55 (0.60–4.01)	1.64 (0.63–4.26)	1.62 (0.63–4.18)
Parity					
Nulliparas	1.00	1.00	1.00	1.00	1.00
Multiparas	1.13 (0.68–1.88)	1.08 (0.65–1.81)	1.11 (0.67–1.85)	1.12 (0.67–1.86)	1.07 (0.64–1.78)
High risk pregnancy*					
no	1.00	1.00	1.00	1.00	1.00
yes	1.09 (0.61–1.96)	1.16 (0.65–2.08)	1.17 (0.66–2.10)	1.12 (0.62–2.01)	1.20 (0.67–2.15)
Weeks of gestation at admission					
>34	1.00	1.00	1.00	1.00	1.00
32–34	1.98 (0.79–4.95)	2.20 (0.88–5.49)	2.06 (0.83–5.14)	2.08 (0.84–5.17)	2.33 (0.94–5.76)
<32	3.21 (1.33–7.75)	3.90 (1.61–9.47)	3.61 (1.50–8.69)	3.35 (1.40–8.01)	4.07 (1.70–9.77)
High risk at admission**					
No	1.00	1.00	1.00	1.00	1.00
Yes	1.32 (0.73–2.37)	1.50 (0.83–2.69)	1.42 (0.79–2.58)	1.26 (0.71–2.25)	1.43 (0.81–2.55)
Level of perinatal care					
Level 1		7.56 (2.36–24.26)			6.54 (2.21–19.40)
Level 2		4.64 (1.48–14.56)			3.68 (1.28–10.59)
Level 3		1.00			1.00
Size of maternity ward (deliveries/yrs)					
<1500			5.27 (1.43–19.44)		
1500–2500			1.73 (0.41–7.35)		
>2500			1.00		
Geographic area					
Ile de France				6.93 (2.12–22.63)	6.83 (2.20–21.16)
Northwest				1.38 (0.41–4.63)	1.42 (0.45–4.47)
Northeast				3.25 (1.11–9.56)	2.49 (0.89–6.94)
Southeast				1.00	1.00
Southwest				5.48 (1.42–21.12)	4.79 (1.33–17.21)
Variance	2.42	2.08	2.16	1.87	1.54
PCV***	reference	0.16	0.12	0.22	0.38

## Discussion

We found that frequency of maintenance tocolysis appears to vary more according to the women’s place of care than according to individual medical risk levels, with a significant maternity unit effect. Part of this effect might be explained by organisational determinants such as level of perinatal care, size of maternity unit, or geographical area. Indeed, the risk of maintenance tocolysis in level 1 and 2 maternity units was at least triple than in level 3 units, up to 5 times higher in small maternity units, and up to 6 times higher in some areas of France.

The EVAPRIMA survey showed that doctors failed to comply with several specific clinical practice guidelines and, in particular, that they prescribed maintenance tocolysis for more than 50% of the women hospitalised for preterm labour. As 6.5% of pregnant women in France are hospitalised for preterm labour each year, 52 000 women might be exposed to a treatment with no proven efficacy and potential side effects. Identifying the factors associated with the prescription of maintenance tocolysis seems important for reducing the exposure of so many women to these unnecessary drugs and to improve adhesion to guidelines.

To our knowledge, this is the first study to assess the factors related to maintenance tocolysis in the management of preterm labour. Because the EVAPRIMA study was a prospective observational survey designed specifically to assess practices related to this management, its database provided a unique opportunity to answer questions about the determinants of maintenance tocolysis. Moreover the EVAPRIMA study differs from questionnaires sent to physicians, which can induce many biases (due to high rate of nonresponse) when studying practices. Its prospective design allows being confident with quality of data. The sample of randomly selected maternity units is representative of French maternity units and did not differ from those included in the 2003 French National Perinatal Survey (i.e., all maternity units in France at that time) for level of perinatal care, hospital status, or size. To avoid selection bias, every woman hospitalised in participating centres over the one-month period was included. The missing data rate was low. Finally, to study only women for whom maintenance tocolysis was possible, we excluded 203 women from the initial EVAPRIMA population. Inclusion of these 203 women would have strengthened the associations observed still further (data not shown).

We used logistic multilevel models, i.e., models adapted to the hierarchical data. Standard logistic models assume that all observations are independent, that is, that women with similar individual characteristics have the same probability of receiving maintenance tocolysis regardless of the maternity unit at which they are treated. We considered women and maternity units as two distinct sources of variability. On one level, women varied among one another according to their socio-demographic and obstetric characteristics. Maternity units, with their organisational characteristics, were treated as a second level. For data with this kind of hierarchical structure, multilevel models provide more accurate measures of confidence intervals than standard logistic models.

Various studies have used different definitions and different agents, most often calcium channel blockers and beta-agonists. Treatment can be performed on an outpatient basis or in hospital [11]–[21]. CNGOF defines maintenance tocolysis as the prescription of tocolysis beyond 48 hours of effective tocolysis, regardless of type of drug or mode of administration. Because EVAPRIMA was an observational survey, the case report forms did not provide a strict definition of maintenance tocolysis but rather left it to the physician’s interpretation. Our definition of maintenance tocolysis could be discussed. Results did not change, however, when we conducted sensitivity analyses with different definitions (data not shown).

The rate of maintenance tocolysis was surprisingly high in the EVAPRIMA study but without any reliable data from other countries, we were unable to conclude if this was specific to France. The reason for the continued widespread use of maintenance tocolysis remains a puzzle. It might be because it was used extensively in the 1980s, and that use has continued since then because it is considered harmless and habits are difficult to change. Another hypothesis is that doctors think that this treatment might provide comfort and reassurance to women returning home after hospitalisation for preterm labour. In this case, maintenance tocolysis, by a placebo effect or by reducing symptoms due to uterine contractions, might reduce women’s anxiety. However, How et al. report that maintenance tocolysis does not reduce the number of readmissions and unscheduled maternity unit visits [22].

The higher “treatment” rate among women admitted before 32 weeks of gestation might be explained by greater concern about early preterm labour. However, the fact that maintenance tocolysis was not more frequent among women with high risk pregnancies or high risks at admission suggests that it is prescribed more according to doctors’ habits than according to individual risks.

Maintenance tocolysis rates were higher among women treated in level 1 and 2 maternity units than among those treated in level 3 units. They were also higher in small maternity units. Several hypotheses might explain these results. First, physicians working in level 3 maternity units, which are usually large teaching hospitals, might be more sensitive to evidence based medicine and their practices thus more consistent with guidelines. Secondly, the concern about preterm delivery might vary with the level of perinatal care. Thus, doctors in level 3 maternity units are much more familiar with preterm labour and therefore less apprehensive about it after an effective acute tocolysis.

Finally the rates of maintenance tocolysis differ between regions of France. Professors in teaching hospitals and other leaders in local and regional obstetrics communities might be responsible for differences in practice between the regions, through teaching and communications at conferences.

Differences observed between maternity units for maintenance tocolysis raise the question of the dissemination and application of clinical guidelines. Few published studies have evaluated the implementation of guidelines in obstetrics. However, several studies have already shown in other medical and surgical disciplines that the application of guidelines is related to both organisational and individual factors [23]–[25]. In these studies, teaching hospitals and hierarchical structure of the ward are associated with higher guideline application, consistent with our findings. Moreover, within a given ward, guideline application depends on doctors’ awareness of their existence, their access to them, their knowledge of them, and their opinions about them [23]. For our study, we did not have any specific information on physicians’ characteristics.

Other factors, not mentioned in our study because they were not available in our database, could also explain some of the maternity unit effect. Status of doctor, i.e junior or consultant, medical specialty or the country of training could influence the rate of maintenance tocolysis. Also on an organisational level, the difficulty of access to the maternity unit or its distance from a level 3 maternity unit could influence the rate of maintenance tocolysis.

By understanding and identifying the reasons associated with the difficulty in applying guidelines, we hope to encourage discussion among health professionals and raise awareness of the need to keep up to date on the literature and guidelines. To improve guideline adhesion for maintenance tocolysis, the guidelines on management of preterm labour appears to require renewed dissemination and publicity in level 1 and 2 maternity units, in small maternity units, and in some French geographic areas. These guidelines should emphasize the lack of efficacy and the possible side effects of this treatment.

### Conclusion

Maintenance tocolysis is widely prescribed in France, regardless of the maternal characteristics at admission for preterm labour. The frequency of this practice appears to vary mostly according to habits in different maternity units, for we found a significant maternity unit effect. Some of this variability between maternity units can be explained by organisational characteristics such as level of perinatal care and geographic area. Broader dissemination of these results might improve adhesion to clinical guidelines.

### Details of Ethics Approval

EVAPRIMA survey received ethics approval from the following institutional ethics committee: Comité Consultatif sur le traitment en matière de Recherche dans le domaine de la santé (CCTIRS) (approval on the 14th april 2005 file n°05.128) and Comission Nationale de l’Informatique et des libertés (CNIL approval on th 7th september 2005 n° 905388).

## Supporting Information

Appendix S1 **Collaborators and participating centres of the EVAPRIMA study.**
(DOC)
