# Supplementary material for: Individual and Organisational Determinants Associated with Maintenance Tocolysis in the Management of Preterm Labour: A Multilevel Analysis
Source: PLoS One. 2012 Dec 13;7(12):e50788. doi: 10.1371/journal.pone.0050788 (PMC3521755; doi:10.1371/journal.pone.0050788)
Supplement: Appendix S1 — Collaborators and participating centres of the EVAPRIMA study. (DOC) [file pone.0050788.s001.doc]

**List of collaborators and participating centres**

Clinique Mutualiste d'Amberieu-01 Ambérieu en Bugey (Dr Planche, Melle Battut), Polyclinique Saint Claude-02 St Quentin (Mme Lambert, Mme Mercier), Centre Hospitalier des Escartons-05 Briançon (Dr Millet), Clinique Saint George-06 Nice (Dr Geraudie, M. Koubi), CHU-06 Nice (Pr Bongain, Mr Delotte), Hôpital Manchester-08 Charleville Mézières (Dr Mereb, Mr Terrosi), Centre Hospitalier Ariège Couserans-09 Saint Girons (Dr Delchambre), Centre Hospitalier-10 Troyes (Dr Brissart, Dr Levert, Mme Durier), Centre Hospitalier Edmond Garcin-13 Aubagne (Dr Savelli), Hôpital-13 Salon de Provence (Dr Raini, Mme Mojard), Centre Hospitalier-13 Martigues (Dr Magnin, Dr Lavergne), Hôpital Clemenceau-14 Caen (Pr Dreyfus, C. Bouet), Centre Hospitalier-17 Rochefort (Dr Dia, Mme Thomas), Hôpital Jacques Cœur-18 Bourges (Dr Ledoux, Dr Portal, Dr Kacem), Clinique Saint Germain-19 Brive la Gaillarde (Mme Blondel), Centre Hospitalier-19 Tulle (Dr Raffi, Dr Assaf), Clinique Chirurgicale Docteur Maymard-20 Bastia (Dr Pinelli), Hôpital-20 Bastia (Dr Colombani, Mme Giannecchini), Centre Hospitalier Général René Pleven-22 Dinan (Dr Chaumet), Polyclinique de Franche Comte-25 Besançon (Dr Hsiung, Mme Malpesa), Hôpital Général-25 Pontarlier (Dr Boyadjian, Mme Zardet), Centre Hospitalier-27 Vernon (Dr Fernet, Mme Aufortdiaz), Centre Hospitalier Site de Louis Pasteur-28 Chartres (Dr Guilbaud, Mme Le Quérec), Polyclinique du Grand Sud-30 Nîmes (Dr Dumontier, Mme Da Silva), Centre Hospitalier-30 Ales (Dr Henry, Dr Khatou), Clinique de L'union-31 St Jean (Dr Bournazeau), Hôpital Joseph Ducuing-31 Toulouse (Dr Charasson, Dr Roberto), Hôpital Jean Hameau-33 La Teste de Buch (Dr Maubaret, Mme Fayard), Hôpital Pellegrin-33 Bordeaux (Pr Horovitz, Pr Dallay, Mr Cardinot, Dr Hamadou-Ousseini, Mme Defaud), Centre Hospitalier-35 Redon (Dr Ladure, Mme Boulanger), Clinique Saint François-36 Châteauroux (Mme Soulé), Centre Hospitalier-36 Le Blanc (Dr Fayad, Mme Desmazieres), Hôpital Bretonneau-37 Tours (Pr Perrotin, Mme Stoeri), Clinique Mutualiste des Eaux Claires-38 Grenoble (Dr Reynaud, Mme Jeannin), Clinique Saint Charles-38 Roussillon (Dr Bert, Dr Perrot, Mme Peirano), Centre Hospitalier-38 Voiron (Dr Giard, Dr Hammouni Houria), Hôpital Louis Pasteur-39 Dole (Dr Barbier, Mme Legentil), Centre Hospitalier Général Maternité de la Ferté-39 Lons Le Saunier (Dr Schwetterle, Mme Dole), Hôpital Pierre Dezarnaulds-45 Gien (Dr Elleuch, Dr Negabi), Polyclinique des Longues Allées- 45 St Jean de Braye (Dr Chave, Mme Brunet), Clinique de l'Anjou Site St Louis-49 Angers (Mme Cloarec), Centre Hospitalier Auban Moet-51 Epernay (Dr Geffroy), Centre Hospitalier Général-52 Langres (Dr Ognong, Dr Geffroy), Hôpital Saint Nicolas-55 Verdun (Dr Pannequin, Mme Bastien), Clinique du Ter-56 Ploemeur (Dr Lagadec, Dr Josset, Mme Mirebaut), Centre Hospitalier du Centre Bretagne-56 Pontivy (Dr Vallée, Mme Ferrand), Clinique Saint Nabor-57 Saint Avold (Dr Benneton, Mme Padoin), Hôpital du Parc-57 Sarreguemines (Dr Sondag), Hôpital Clinique Claude Bernard-57 Metz (Dr Adamy, Mme Swaeles), Hôpital Notre Dame de Bon Secours-57 Metz (Dr Manin, Dr Nseir), Clinique Du Nohain-58 Cosne sur Loire (Dr Bonnemaison, Mme Vilaire), Centre Hospitalier-59 Cambrai (Dr Alrayes), Clinique Lille Sud-59 Lesquin (Mme Tuffier, Mme Smague), Polyclinique du Val de Lys-59 Tourcoing (Dr Bouche, Mme Taniel), Clinique Sainte Marie-59 Cambrai (Dr Devoldère, Mme Bertiaux), Hôpital Général-60 Clermont de l'Oise (Dr Kingue-Ekollo), Hôpital Région Saint Omer-62 Helfaut (Dr Pauchet), Centre Hospitalier-62 Calais (Dr Beral, Dr Souhar), Centre Hospitalier Général-65 Lourdes (Dr Benabi), Centre Hospitalier-66 Perpignan (Dr Bachelard, Mme Cayrac), Centre Medico-chirurgical-Obstétrical-67 Schiltigheim (Dr Favre, Dr Vayssiere, Mme Psychogios), Centre Hospitalier Général-67 Sélestat (Dr Grall, Mme Clauss), Clinique Saint Sauveur-68 Mulhouse (Dr Grenet, Mme Winnlen), Hôpital de la Croix Rousse-69 Lyon (Pr Rudigoz, Mme Lagorce), Clinique Monplaisir-69 Lyon (Mme Curtet), Polyclinique Pasteur-69 St Priest (Mme Berlioz), Hôpital Edouard Herriot-69 Lyon (Pr Gaucherand, Mme Venaruzzo), Centre Hospitalier-71 Autun (Dr Hibelot, Mme Compagnon), Clinique de l’Espérance-74 Cluses (Dr Nahrat, Mme Autissier), Centre Hospitalier-74 Annecy (Dr Tardif, Dr Arnould), Hôpital Intercommunal Sud Leman Valserine-74 St Julien en Genevois (Dr Toccanier, Dr Ghosn), Institut Mutualiste Montsouris Jourdan-75 Paris (Dr Cohen, Mme Delahaye), Hôpital Tenon-75 Paris (Pr Uzan, Dr Sakr), Hôpital Cochin-75 Paris (Pr Goffinet, Mme Onquierts), Clinique du Petit Colmoulins-76 Harfleur (Dr Capella), Groupe Hospitalier du Havre-76 Le Havre (Dr Schweitzer, Dr Talbot), Centre Hospitalier Général-76 Fécamp (Dr Baril), Hôpital-77 Meaux (Dr Michel, Dr Wipff, Mme Pernet), Hôpital Prive de L'ouest Parisien-78 Trappes (Dr Vielh), Centre Hospitalier-80 Péronne (Dr Puech, Mme Daudre), Hôpital Général de Castres Mazamet-81 Castres (Dr Fabries, Mme Sonzogni), Clinique Saint Jean-83 Toulon (Dr Thomas, Dr Noujaim), CHI-83 Toulon (Dr Dausset), Centre Hospitalier de Cavaillon Lauris-84 Cavaillon (Dr Beaupretre, Mme Cherubini), Centre Hospitalier Louis Giorgi-84 Orange (Dr Galli, Mme Bobert), Polyclinique Sainte Anne-86 Châtellerault (Dr Boisselier), Centre Hospitalier Camille Guerin-86 Châtellerault (Dr Godard, Mme Dabilly), Clinique de l'Arc en Ciel-88 Epinal (Dr Orefice, Mme Du Sartz de Vigneulles, Mme Thiriet), Centre Hospitalier Général-88 Neufchâteau (Dr Henry, Mme Macquet), Centre Hospitalier Saint Charles-88 St Die des Vosges (Dr Ringele, Mme Martin), Centre Hospitalier de Belfort Montbeliard-90 Belfort (Dr Terzibachian, Mme Bos), Maternité de L'yvette-91 Longjumeau (Dr Le Thou Viguijen, Mme Howe), Clinique Ambroise Paré-92 Bourg la Reine (Dr Daoud, Mme Lamarche), Clinique Sainte Isabelle-92 Neuilly sur Seine (Dr Meuneux, Mme Mangin), Hôpital Beaujon-92 Clichy (Pr Levardon, Dr El Houarri), Hôpital Max Fourestier-92 Nanterre (Dr De Sarcus, Dr Bentolba), Hôpital Foch-92 Suresnes (Pr Colau, Dr Simon), Centre Hospitalier-92 Neuilly sur Seine (Dr Botto, Dr Jardin), Clinique du Bois d’Amour-93 Drancy (Dr Trentesaux, Dr Chevallier), Hôpital Jean Verdier-93 Bondy (Pr Uzan, Mme Pharisien), Centre Hospitalier Intercommunal-94 Villeneuve Saint Georges (Dr Maria, Mme Starck), Centre Hospitalier Intercommunal-94 Créteil (Pr Painal, Mme Jarjanette), Nouvelle Société-95 Enghien les Bains (Dr Fauck, Mme Songue-Edimo), Clinique Conti-95 L'Isle Adam (Dr Bommel, Dr Lespour), Polyclinique du Lac d'Enghien-95 Soisy sous Montmorency (Dr Vezin, Mme Sanson), Centre Hospitalier-95 Gonesse (Dr Dauptain, Dr Duckat),
